# Effects of fish oil supplementation on glucose control and lipid levels among patients with type 2 diabetes mellitus: a Meta-analysis of randomized controlled trials

**DOI:** 10.1186/s12944-020-01214-w

**Published:** 2020-05-08

**Authors:** Chao Gao, Yang Liu, Yong Gan, Wei Bao, Xiaolin Peng, Qingbin Xing, Huiyu Gao, Jianqiang Lai, Liegang Liu, Zhu Wang, Yuexin Yang

**Affiliations:** 1 Key Laboratory of Trace Element Nutrition of National Health Commission, National Institute for Nutrition and Health, Chinese Center for Disease Control and Prevention, No 29 Nanwei Road, Beijing, 100050 China; 2 School of Public Health, Tongji Medical College, Ministry of Education Key Laboratory of Environment, Hubei Key Laboratory of Food Nutrition and Safety, Huazhong University of Science and Technology, Wuhan, 430030 China; 3 Department of Epidemiology, College of Public Health, Fraternal Order of Eagles Diabetes Research Center, University of Iowa, Iowa, 52242 USA; 4 Shenzhen Nanshan Center for Chronic Disease Control, Shenzhen, 518000 China

**Keywords:** Fish oil, Omega-3 fatty acids, Type 2 diabetes mellitus, Randomized clinical trials, Meta-analysis

## Abstract

**Background:**

Previous studies have yielded inconsistent findings on the role of fish oil in type 2 diabetes mellitus (T2DM). We systematically summarized the available evidence from randomized controlled trials (RCT) and aimed to investigate the effects of fish oil supplementation on glucose control and lipid levels among patients with T2DM.

**Methods:**

A comprehensive literature search was performed in electronic databases (PubMed, ProQuest, Cochrane Library, CNKI, VIP, and Wanfang) to identify all relevant RCTs which were published up to May 31st, 2019. We used Modified Jadad Score system to evaluate the quality of each included RCT. The pooled effects were estimated using random-effects model and presented as standardized mean differences with 95% confidence intervals.

**Results:**

A total of 12 RCTs were included in this meta-analysis. There was no significant difference in glucose control outcomes comparing fish oil supplementation to placebo. The effect size of fasting plasma glucose (FPG) was 0.13 (95% CI: − 0.03 to 0.28, *p* > 0.05). No marked change was observed in fasting insulin (FINS), glycosylated hemoglobin (HbA1c), and HOMA of insulin resistance (HOMA-IR) levels. Fish oil supplementation was associated with a decrease of triglyceride (TG) level by − 0.40 (95%CI: − 0.53 to − 0.28, *p* < 0.05), and an increase of high density lipoprotein (HDL) cholesterol level by 0.21 (95%CI: 0.05 to 0.37, *p* < 0.05). In subgroup analysis, HDL cholesterol level was higher among Asian and low-dose(< 2 g/d n-3 PUFA) subgroups compared to their counterparts (*p* < 0.05). TG level was lower in mid and long duration groups, along with an inconspicuous difference in short duration group.

**Conclusions:**

This meta-analysis shows that among patients with T2DM, fish oil supplementation leads to a favorable blood lipids profile but does not improve glucose control.

## Introduction

Type 2 diabetes mellitus (T2DM) is a long-term metabolic disorder which is characterized by high blood glucose in conditions of insulin resistance and/or insufficient insulin secretion [1]. It is one of the most common chronic diseases in the world, which is also a major risk factor for coronary heart disease (CHD), blindness, kidney failure, and all types of cancer [2]. The global prevalence of diabetes was 422 million at 2014, and it was the seventh leading cause of death in 2016, along with tremendous economic and public health burdens [3]. It has been stated that most of T2DM can be partly prevented by proper diets and healthy lifestyles, which triggered the investigation of specific dietary interventions.

Mainly found in fish oils, eicosapentaenoic acid (EPA) and docosahexaenoic acid (DHA) are marine n-3 polyunsaturated fatty acids (PUFAs) involved in human physiology; received increasing attention in recent years due to its wide range of biological activities. Some studies showed that n-3 PUFAs may reduce the risk of CHD, ischemic stroke, alleviate inflammation and promote healthier cognitive aging [4–9]. For the past few years, animal and cell culture studies have demonstrated that n-3 PUFAs also have beneficial effects on prevention of T2DM through multiple mechanisms including insulin signaling, anti-inflammatory actions, altering cell membrane function, and control the expression of glucose metabolism genes [10]. However, results from human intervention studies have been inconsistent [6, 11–15], which might be attributed to mixed subjects and various intervention measures [16, 17]. Thus, it is imperative to comprehensively evaluate the effects of fish oil supplementation on T2DM patients to inform clinical guidelines [18].

Decrease of triglyceride levels by fish oil supplementation has been confirmed by previous studies [19], but other indicators of blood lipid and glucose control remain inconclusive [20–22]. For instance, a meta-analysis [21] reported that fasting plasma glucose level in type 2 diabetic Asians was increased by 0.42 mmol/L with n-3 PUFAs supplementation, while no change was identified in the whole type 2 diabetic population. However, some aspects included in this study were varied. Supplementation ranged from fish oil, purified omega-3 fatty acids and EPA-E whereas subjects with impaired glucose tolerance subjects were also included. In another study [23], fish oil was associated with lower risk of insulin resistance among metabolic syndrome (MS) participants, but no effect was observed in T2DM patients or healthy people. Although fish oil is one of the most common complementary forms of n-3, complete meta data is still lacking in the ralationship between fish oil and type 2 diabetes. Furthermore, several RCTs have come up with richer conclusions, which could further contribute to the pooled data and allow further investigation into any association between fish oil consumption and T2DM. Therefore, we aimed to conduct a systematic review and meta-analysis to examine the effect of fish oil supplementation on blood glucose control and lipid levels among patients with T2DM.

## Methods

### Literature search

We searched databases of Pubmed, ProQuest, Cochrane Library, CNKI, VIP, Wanfang from the beginning until May 31st 2019. Our comprehensive search was performed using the following key words in combination as both MeSH terms and text words: [fish oil or omega-3 fatty acids or n-3 PUFA or Docosahexaenoic Acids (DHA) or Eicosapentaenoic Acid (EPA)] and [TG or TC or HDL cholesterol or LDL cholesterol or fasting plasma glucose (FPG) or fasting plasma insulin (FINS) or glycosylated hemoglobin (HbA1c) or HOMA of insulin resistance (HOMA-IR)] and [Type 2 diabetes mellitus or T2MD] and [Randomized Controlled Trials or RCT]..

### Search selection

Two separate investigators examined both titles and abstracts to determine the relevance of RCTs. Full articles were retrieved for further assessment following the inclusion criteria: 1) human RCTs (either parallel or crossover design) with fish oil supplementation intervention; 2) the study population was T2DM patients; 3) the control group received placebos without n-3 fatty acid elements. 4) the study reported outcomes including TG, TC, HDL cholesterol, LDL cholesterol, fasting plasma glucose (FPG), fasting plasma insulin (FINS), glycosylated hemoglobin (HbA1c), HOMA of insulin resistance (HOMA-IR) at baseline and the end of trial. Interventions with fish diet, pure n-3 fatty acids or other multifactorial intervention were excluded. Articles with incomplete information, poor quality and repeated populations were also excluded. RCTs with serious complications of type 2 diabetes were also removed. In addition, crossover design studies without data of the first period of intervention were excluded. When there were disagreements about the study inclusion, we consulted a third author.

### Data extraction and quality evaluation

Two investigators (C.G and Y.L) conducted a data extraction form respectively to collect the information, which included the first author’s name, year of publication, study design, sample size, participants’ characteristics, intervention details (dosage, type, frequency, and duration), placebo, outcomes index. Sample size (*n*), mean and standard deviation (SD) were extracted from both intervention group and control group at baseline and the end of trial. When data were reported as standard errors (SEs), interquartile range (IQR), or 95%CI, SDs were calculated by equations $SD= SE\times \sqrt{\mathrm{n}}$, *SD* = *IQR* ÷ 1.35, $SD=\sqrt{n}\times \left( upper- lower\right)/2\times t$, respectively (The value 't' is the degree of freedom). If a study reported outcome index at two sections or two doses, we regarded them as two independent studies. When some data were not directly reported in the article, the original author was contacted to obtain the relevant information. The methodology quality of RCTs was graded by Modified Jadad Score system. Studies with a score of 1–3 were considered as low quality whereas a score of 4–7 as high quality.

### Statistical analysis

Data were analyzed by STATA 12.0 statistical software. The pooled effects were estimated in a meta-analysis using random-effects model and presented as standardized mean differences (SMD) with 95% confidence intervals (95%CI). Heterogeneity was assessed using I^2^ tests with significance value set at *p* < 0.05. Risk of publication bias was evaluated by the Egger’s and Begg’s tests with p < 0.05 as significant bias. Influence analysis method was used to perform the sensitivity test. To analyze the potential sources of heterogeneity, subgroup analyses were undertaken according to race (Asian, America and European), intervention duration (≤1 month, 1-3 months, > 3 months), and dose of n-3 PUFA existed in fish oil (< 2 g/d for low dose, ≥2 g/d for high dose). *P* value< 0.05 were set to be statistically significant.

## Results

### Study identification

**Fig. 1:**
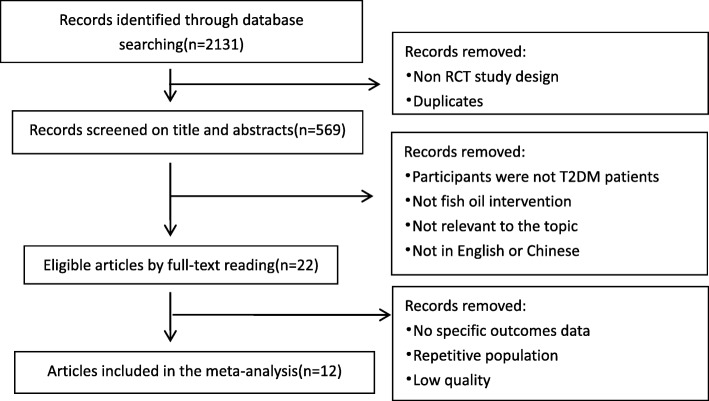
Flow chart on the articles selection process

We retrieved 2131 potentially eligible papers from electronic database searching. After removing duplicates and improper designs by screening title and abstracts, 569 papers remained. We then excluded studies which were not conducted in T2DM participants, did not use fish oil intervention, not relevant to the topic, and not in English or Chinese through full-text reading. Among the 22 remaining articles, 8 studies had no specific outcomes data, one study was regarded as low quality and 2 studies were conducted with the same population. For the two articles based on the same population, the one with more data was chosen. Finally, 12 RCTs met the eligibility criteria and were included in the meta-analysis. The details are shown in Fig. 1.

### Study characteristics

The main characteristics of the studies included are shown in Table 1. The 12 RCTs included 2 single-blind RCTs and 10 double-blind RCTs which were published between Jan 1st 1990 and May 31st 2019. There were 820 adult participants involved and they were all diagnosed as T2DM patients. Among these RCTs, 6 studies were from Asia, 3 studies were from Europe, the other 3 articles were from North America and Brazil. Duration of the interventions varied from 3 weeks to 6 months. The doses of n-3 PUFA existed in fish oil are ranged from 0.3 g/d to 10.08 g/d.

**Table 1:** Characteristics of the included studies

Study (year)	Design	Type of Patient	Location	Number	Gender	Age	Duration	Study arms	Dose (g/d)	Control
Wang 2017 [24]	RCT	T2DM	China	156	M51 F105	≥60	3 m,6 m	Fish oil	4	Corn oil
Gao 2017 [25]	RCT	T2DM	China	67	M30 F37	M:40–70 F:menopause-70	3 m,6 m	Fish oil	4	Corn oil
Jacobocejudo 2017 [14]	RCT	T2DM	Mexico	65	M15 F50	25–60	6 m	Fish oil	2.8	Cornstarch
Mansoori 2015 [26]	RCT	T2DM	Iran	68		30–70	2 m	Fish oil	2.4	Paraffin oil
Zheng 2014 [27]	RCT	T2DM	China	36		M:40–80 F:menopause-80	6 m	Fish oil	4	Corn oil
Crochemore 2012 [28]	RCT	T2DM	Brazil	41	F41	60.64 ± 7.82	1 m	Fish oil	1.5/2.5	Placebo
Fakhrzadeh 2010 [29]	RCT	T2DM	Iran	124		74.81 ± 9.44	6 m	Fish oil	1	Medium chain triglycerides oil
Wong 2010 [30]	RCT	T2DM	China	97	M43 F54	Control:59.0 ± 9.3 Fish oil:61.2 ± 9.0	3 m	Fish oil	4	Olive oil
Kabir 2007 [31]	RCT	T2DM	France	26	F26	40–60	2 m	Fish oil	3	Paraffin oil
Morgan 1995 [32]	RCT	T2DM	USA	40	M18 F22	53.9 ± 7	6w,12w	Fish oil	9/18	Corn oil
Pelikanova 1993 [33]	RCT	T2DM	Czech	20	M20	40–60	3w	Fish oil	15 ml	Saline solution
Hendra 1990 [34]	RCT	T2DM	UK	80	M55 F25	55.9 ± 11.5	6 W	Fish oil	10	Olive oil

### Quality assessment

As Table 2 shown, results from Modified Jadad Score system revealed that 6 of the included trials were low quality, while the other 6 trials were high quality.

**Table 2:** Modified Jadad Score form

Study (year)	Randomization	Concealment of allocation	Double blinding	Withdrawals and dropouts	Score
Wang 2017 [24]	2	1	2	1	6
Gao 2017 [25]	2	1	2	0	5
Cejudo 2017 [14]	1	1	0	0	2
Mansoori 2015 [26]	1	1	1	0	3
Zheng 2014 [27]	1	2	2	0	5
Crochemore 2012 [28]	1	1	0	0	2
Fakhrzadeh 2010 [29]	1	1	1	1	4
Wong 2010 [30]	2	2	2	1	7
Kabir 2007 [31]	1	1	1	1	4
Morgan 1995 [33]	1	1	1	0	3
Pelikanova 1993 [34]	1	1	1	0	3
Hendra 1990 [35]	1	1	1	0	3

### Meta-analysis

**Fig. 2:**
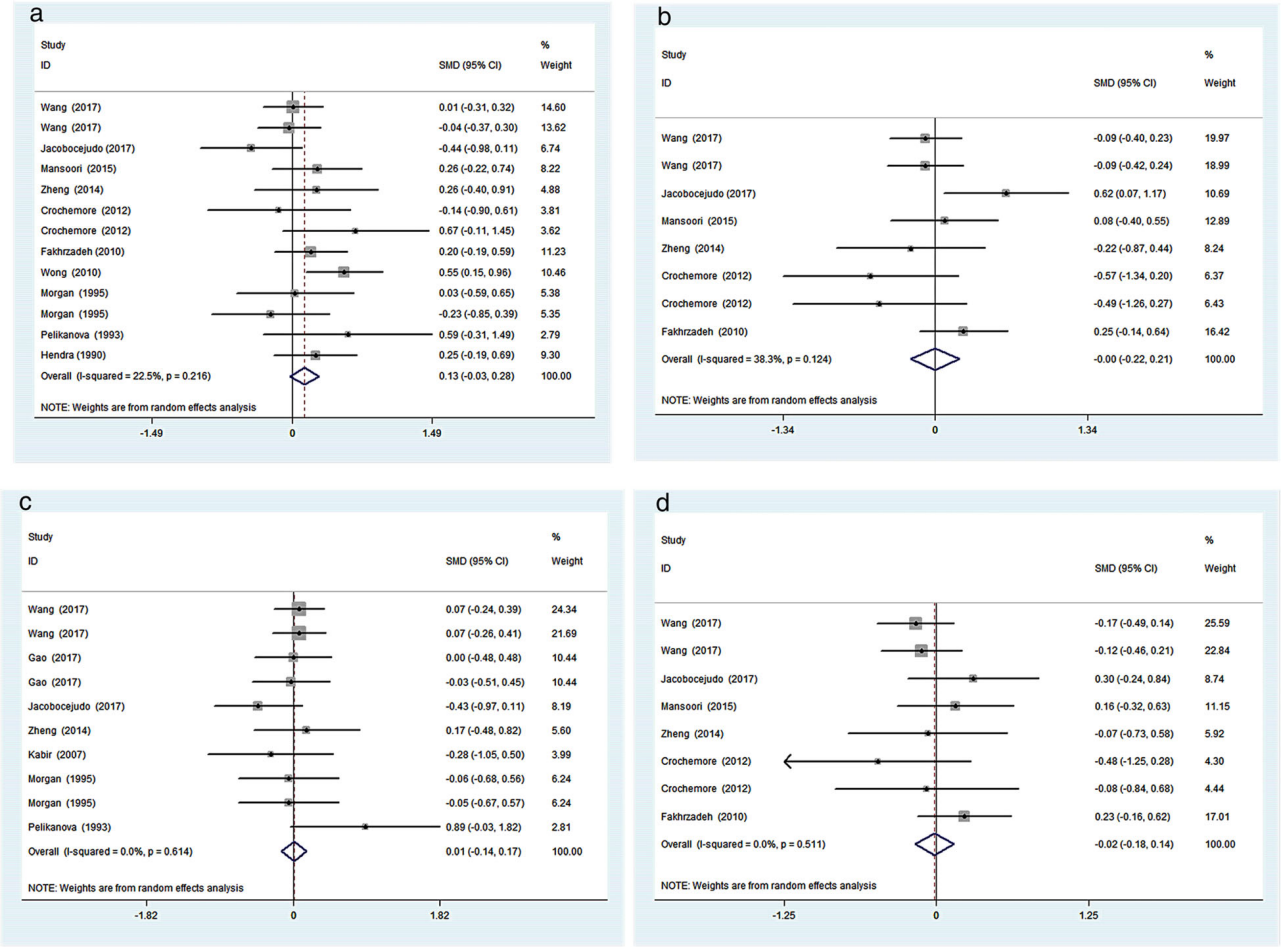
Effects of fish oil on FPG(**a**), FINS(**b**), HbA1c(**c**), HOMA-IR(**d**)

**Fig. 3:**
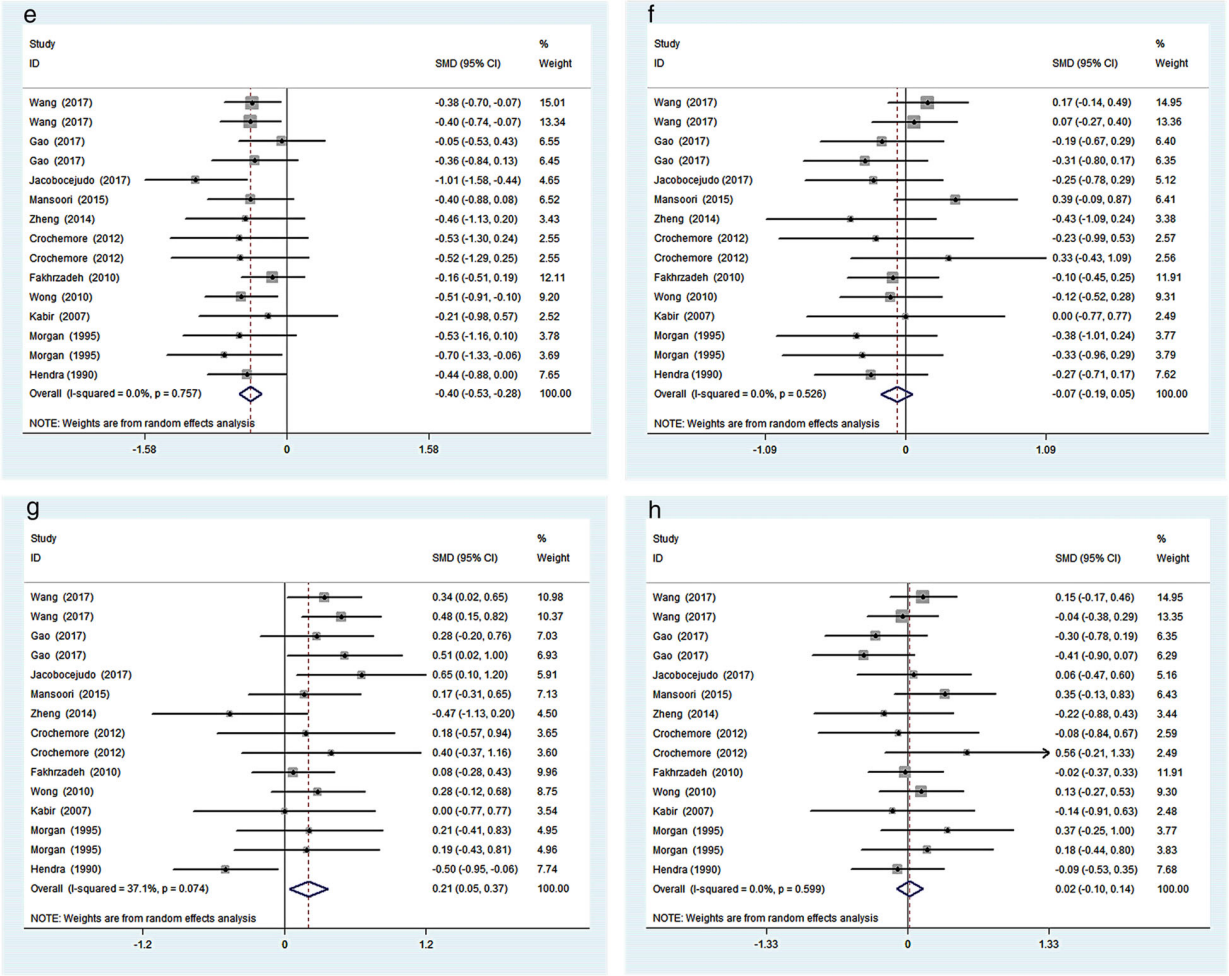
Effects of fish oil on TG(**e**), TC(**f**), HDL cholesterol(**g**), LDL(**h**) cholesterol

Among the included studies, 13 studies reported data on FPG, 8 reported data on FINS, 10 reported data on HbA1c and 8 reported data on HOMA-IR. Meanwhile, 15 studies reported data on lipid parameters. The pooled analysis showed that there was no significant difference in glucose control outcomes comparing fish oil supplementation to placebo. However, compared to placebo, fish oil supplementation significantly decreased TG level by − 0.40 (95%CI: − 0.53 to-0.28, I^2^ = 0%, *p* < 0.05), and increased HDL cholesterol level by 0.21 (95%CI: 0.05 to 0.37, I^2^ = 37.1%, *p* < 0.05). There were no significant changes in TC or LDL cholesterol level (Figs. 2 and 3).

### Subgroup analyses

We undertook subgroup analyses according to race, intervention duration and dose of n-3 PUFA existed in fish oil supplementation. Table 3 showed the data from trails with Asian and US/European subjects. No marked differences were identified in glycemic parameters. Data of TG levels displayed protective effects on both populations, but this change was more obvious in US/European population (SMD: -0.58, 95%CI: − 0.81 to − 0.34, *p* < 0.05). We also found that fish oil supplementation increased HDL cholesterol level in the Asian subgroup (SMD: 0.26, 95%CI: 0.10 to 0.43, *p* < 0.05) whereas the above was not observed in US/European subjects. Non-significant results were observed for the other lipid parameters assessed.

**Table 3:** Subgroup analysis of fish oil supplementation and placebo by race

Outcome	No. of studies	Overall effect		Heterogeneity(%)
		Effect size(95%CI)	***p***	I^***2***^
**FPG**	13	0.13(− 0.03,0.28)	0.11	22.5
Asian	6	0.17(− 0.01,0.35)	0.07	18.5
US/European	7	0.05(− 0.24,0.34)	0.72	31.3
**FINS**	8	− 0.00(− 0.22,0.21)	0.98	38.3
Asian	5	−0.01(− 0.18,0.17)	0.96	0
US/European	3	−0.11(− 0.94,0.72)	0.80	76.8
**HbAc1**	10	0.01(−0.14,0.17)	0.88	0
Asian	5	0.06(− 0.13,0.24)	0.55	0
US/European	5	−0.01(− 0.44,0.30)	0.71	34.5
**HOMA-IR**	8	−0.02(− 0.18,0.14)	0.82	0
Asian	5	−0.02(− 0.20,0.15)	0.78	0
US/European	3	−0.02(− 0.48,0.44)	0.93	27.8
**TG**	15	−0.40(− 0.53,-0.28)	0	0
Asian	8	−0.34(− 0.48,-0.19)	0	0
US/European	7	−0.58(− 0.81,-0.34)	0	0
**TC**	15	−0.07(− 0.19,0.05)	0.28	0
Asian	8	−0.02(− 0.18,0.14)	0.81	15.1
US/European	7	−0.21(− 0.44,0.02)	0.08	0
**HDL cholesterol**	15	0.21 (0.05,0.37)	0.01	37.1
Asian	8	0.26 (0.10,0.43)	0.00	20
US/European	7	0.14(−0.20,0.47)	0.42	49.2
**LDL cholesterol**	15	0.02(−0.10,0.14)	0.72	0
Asian	8	−0.01(− 0.17,0.15)	0.91	13.8
US/European	7	0.09(−0.14,0.32)	0.44	0

Abbreviations: *FPG* fast plasma glucose; *FINS* fast insulin; *HbA1c* glycosylated hemoglobin; *HOMA-IR* HOMA of insulin resistance; *TG* triglyceride; *TC* total cholesterol; *HDL cholesterol* high density lipoprotein cholesterol; *LDL cholesterol* low density lipoprotein cholesterol

Table 4 presented the data according to the different terms (short-term duration (≤1 month), mid-term duration (1–3 months), and long-term duration (> 3 months)) of intervention. Variations of TG level only appeared in mid-term (SMD: -0.40, 95%CI: − 0.56 to − 0.23, *p* < 0.05) and long-term (SMD: -0.43, 95%CI: − 0.68 to − 0.17, *p* < 0.05) subgroups, indicating that the duration of intervention played a dramatic role in the change in TG levels. While short-term intervention of less than 1 month might not make sense. No statistic differences were discovered for other outcomes.

**Table 4:** Subgroup analysis of fish oil supplementation and placebo by intervention duration

Outcome	No. of studies	Overall effect		Heterogeneity(%)
		Effect size(95%CI)	***p***	I^***2***^
**FPG**	13	0.13(− 0.03,0.28)	0.11	22.5
≤1 month	3	0.35(−0.18,0.88)	0.19	21.9
1-3 months	6	0.17(−0.04,0.38)	0.11	23.0
> 3 months	4	0.00(−0.26,0.27)	0.99	27.6
**FINS**	8	−0.00(− 0.22,0.21)	0.98	38.3
≤1 month	2	−0.53(−1.08,0.01)	0.06	0
1-3 months	2	−0.04(− 0.30,0.23)	0.79	0
> 3 months	4	0.14(−0.19,0.47)	0.41	51.7
**HbAc1**	10	0.01(−0.14,0.17)	0.88	0
≤1 month	1	0.89(− 0.03,1.82)	0.06	–
1-3 months	5	− 0.00(− 0.22,0.22)	1.00	0
> 3 months	4	−0.03(− 0.26,0.20)	0.81	0
**HOMA-IR**	8	−0.02(− 0.18,0.14)	0.82	0
≤1 month	2	−0.28(− 0.82,0.26)	0.31	0
1-3 months	2	− 0.06(− 0.37,0.25)	0.71	22
> 3 months	4	0.06(− 0.16,0.28)	0.59	0
**TG**	15	−0.40(− 0.53,-0.28)	0	0
≤1 month	2	−0.53(−1.07,0.02)	0.06	0
1-3 months	8	−0.40(− 0.56,-0.23)	0.00	0
> 3 months	5	−0.43(− 0.68,-0.17)	0.00	36.8
**TC**	15	−0.07(− 0.19,0.05)	0.28	0
≤1 month	2	0.05(− 0.50,0.60)	0.86	4.7
1-3 months	8	−0.05(− 0.23,0.14)	0.61	16.7
> 3 months	5	−0.12(− 0.32,0.07)	0.20	0
**HDL cholesterol**	15	0.21 (0.05,0.37)	0.01	37.1
≤1 month	2	0.29(−0.25,0.83)	0.29	0
1-3 months	8	0.14(−0.07,0.35)	0.20	33.3
> 3 months	5	0.29(−0.04,0.61)	0.09	60.6
**LDL cholesterol**	15	0.02(−0.10,0.14)	0.72	0
≤1 month	2	0.23(−0.40,0.86)	0.47	26.2
1-3 months	8	0.09(−0.08,0.25)	0.29	0
> 3 months	5	−0.10(− 0.29,0.10)	0.33	0

Abbreviations: *FPG* fast plasma glucose; *FINS* fast insulin; *HbA1c* glycosylated hemoglobin; *HOMA-IR* HOMA of insulin resistance; *TG* triglyceride; *TC* total cholesterol; *HDLcholesterol* high density lipoprotein cholesterol; *LDL cholesterol* low density lipoprotein cholesterol; −, not available

Table 5 showed the data of subgroup analysis with low-dose (< 2 g/d) and high-dose (≥2 g/d) of n-3 PUFA existed in fish oil supplementation. A significant reduction of TG level was observed in both low-dose (SMD: -0.36, 95%CI: − 0.57 to − 0.15, *p* < 0.05) and high dose (SMD: -0.45, 95%CI: − 0.62 to − 0.29, *p* < 0.05) subgroups. The results also demonstrated that HDL level was increased only in the low-dose subgroup (SMD: 0.27, 95%CI: 0.08 to 0.45, *p* < 0.05). There was no significant difference in glycemic index followed by this subgroup analysis.

**Table 5:** Subgroup analysis of fish oil supplementation and placebo by dose of n-3 PUFA

Outcome	No. of studies	Overall effect		Heterogeneity(%)
		Effect size(95%CI)	***p***	I^***2***^
**FPG**	13	0.13(−0.03,0.28)	0.11	22.5
< 2 g	5	0.10(−0.23,0.42)	0.56	42.2
≥2 g	8	0.14(−0.04,0.32)	0.13	17.8
**FINS**	8	−0.00(− 0.22,0.21)	0.98	38.3
< 2 g	5	−0.06(− 0.32,0.44)	0.76	56.6
≥2 g	3	−0.10(− 0.32,0.11)	0.36	0
**HbAc1**	10	0.01(−0.14,0.17)	0.88	0
< 2 g	4	−0.15(− 0.42,0.12)	0.28	0
≥2 g	6	0.10(− 0.10,0.28)	0.34	0
**HOMA-IR**	8	−0.02(− 0.18,0.14)	0.82	0
< 2 g	5	0.13(−0.11,0.36)	0.29	0
≥2 g	3	−0.14(− 0.36,0.07)	0.20	0
**TG**	15	−0.40(− 0.53,-0.28)	0.00	0
< 2 g	8	−0.36(− 0.57,-0.15)	0.00	16.5
≥2 g	7	−0.45(− 0.62,-0.29)	0.00	0
**TC**	15	− 0.07(− 0.19,0.05)	0.28	0
< 2 g	8	− 0.07(− 0. 25,0.12)	0.48	0
≥2 g	7	−0.08(− 0.25,0.09)	0.37	8.8
**HDL cholesterol**	15	0.21 (0.05,0.37)	0.01	37.1
< 2 g	8	0.27 (0.08,0.45)	0.00	0
≥2 g	7	0.11(−0.18,0.40)	0.44	64.7
**LDL cholesterol**	15	0.02(−0.10,0.14)	0.72	0
< 2 g	8	−0.03(− 0.23,0.18)	0.80	17.5
≥2 g	7	0.06(−0.10,0.22)	0.45	0

Abbreviations: *FPG* fast plasma glucose; *FINS* fast insulin; *HbA1c* glycosylated hemoglobin; *HOMA-IR* HOMA of insulin resistance; *TG* triglyceride; *TC* total cholesterol; *HDL cholesterol* high density lipoprotein cholesterol; *LDL cholesterol* low density lipoprotein cholesterol

### Sensitivity analysis and publication bias

**Fig. 4:**
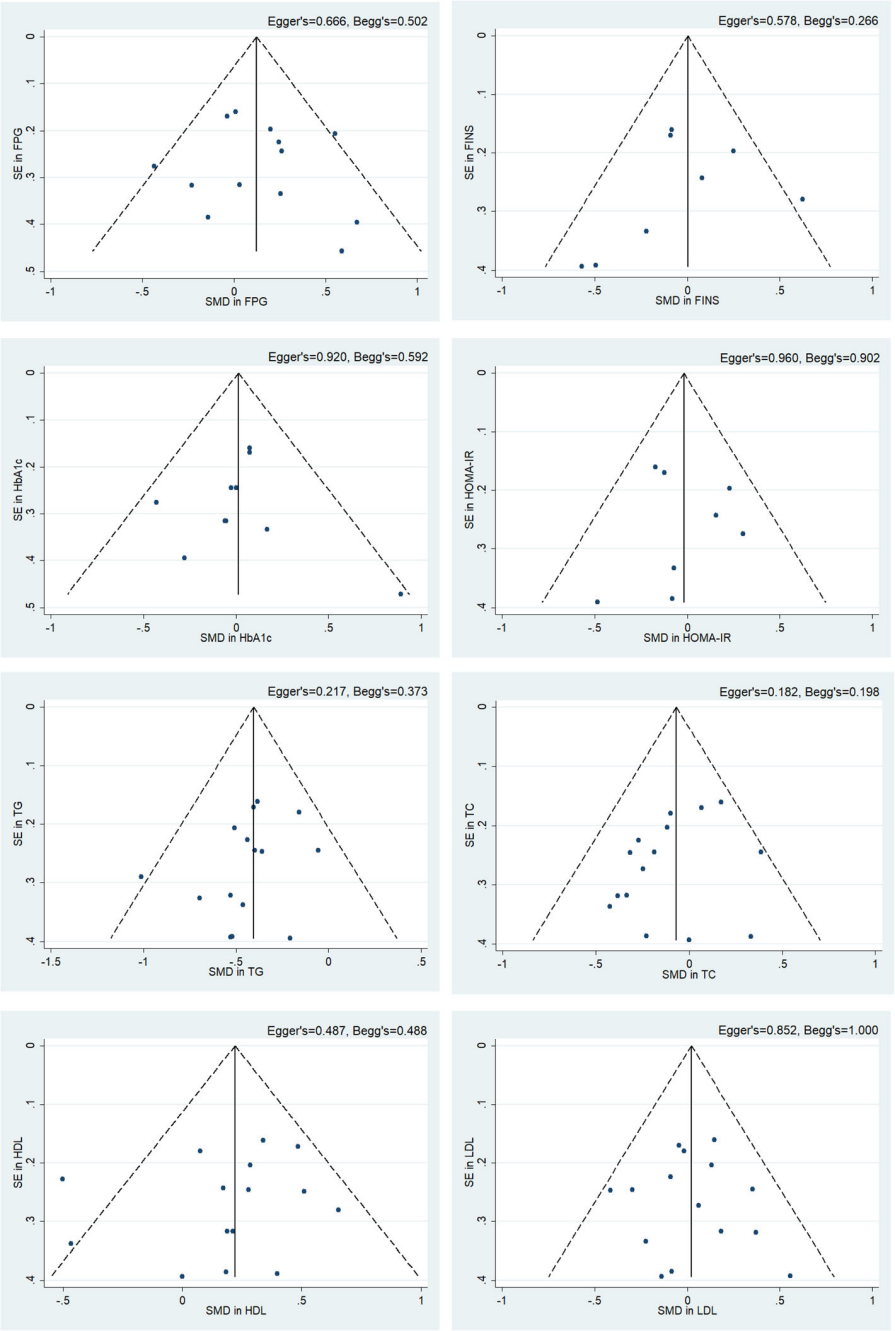
Funnel plots assessing publication bias. Publication bias and small-study effects were assessed using Egger’s and Begg’s test and presented as *P* values

The sensitivity analysis plot of each observed indicators presented no effect size being out of the 95%CI and the combined 95%CI, which indicated that our results were stable and reliable. Results of the publication bias tests stated that there was no potential publication bias, with *P*-value of Egger’s test for each parameter was 0.217 (TG), 0.182 (TC), 0.487 (HDL cholesterol), 0.852 (LDL cholesterol), 0.666 (FPG), 0.578 (FINS), 0.920 (HbA1c), 0.960 (HOMA-IR), respectively. The funnel plot, Egger’s and Begg’s values were shown in Fig. 4.

## Discussions

This meta-analysis of 12 RCTs on T2DM patients found that TG level decreased significantly with fish oil supplementation. Fish oil intervention was also effective in elevating HDL cholesterol level. Previous studies have shown that a reduced TG level and elevated HDL cholesterol level are associated with lower risk of atherosclerosis and cardiovascular disease [18]. In the subgroup analysis, a more obvious decrease in TG level was observed in US/European subjects compared to Asians subjects. On the other hand, HDL cholesterol level in Asians presented a statistical increase but was not shown in US/European subjects. The above indicated a non-negligible role of racial difference which suggest further exploration on genomics. With respect to potential mechanisms of action, fish oil was associated with peroxisome proliferator-activated receptors (PPAR). Genetic variants at PPARG associated with T2DM were only found in GWAS of Europeans but not in East Asians [35]. The effects of n-3 PUFA on blood lipids in type 2 diabetic patients may vary according to the genetic variations of CD36, NOS3 and PPAR-gamma genes, and personalized dietary recommendations based on certain genetic components to improve blood lipid profile may be extremely effective for n-3 PUFA intake [36].

We also found that changes in TG level appeared only when the intervention period was above 1 month. This result was consistent with a previous study which reported that 4 weeks were needed for serum PUFA levels to reach equilibrium [37]. It is worth mentioning that the results may also be due to the relatively small number of short-term intervention trials included in this paper. Besides that, the n-3 PUFA showed resultful effects on both TG and HDL cholesterol level in low dose group. However, when the intake is above 2 g/d, n-3 PUFA had a more significant effect on TG level, which did not seen in HDL cholesterol level. Since there are few literature studies on the relationship between n-3 PUFA intake and HDL cholesterol level, and in the limited trials included in this meta-analysis, the intake of n-3 PUFA even reached more than 10 g, more in-depth studies are still needed in this regard. In Ree’s study [38], the necessary dose of eicosapentaenoic acid to achieve an anti-inflammatory was reported to be at least 2 g/d, which can provide reference for future research.

Although there was no significant association between fish oil supplementation and blood glucose control levels in our study, conflicting results have emerged in other studies. For example, a previous meta-analysis of 20 RCTs using omega-3 fatty acids as supplementation in T2DM patients discovered an increase in FPG level by 0.42 mmol/L (95%CI: 0.058 to 0.781 mmol/L, *p* < 0.05) in Asians whilst no change was observed in the whole T2DM population [21]. Conversely, another meta-analysis of 24 observational cohort studies [39] showed that marine omega-3 fatty acids could significantly reduce T2DM risk in Asians (RR: 0.87, 95%CI: 0.79 to 0.96). One possible reason mentioned in the previous article is that most of the RCT intervention lasted less than 12 weeks which may cause null effect on glucose control [21]. However, according to our subgroup analysis, no significant effect was observed even when intervention was more than three months. Another possible explanation is that the high differentiation of gene loci in different race of T2DM patients affects the incidence of T2DM [39, 40]. Studies have shown that differences in T2DM loci in East Asian and European populations directly lead to different responses to n-3 PUFA exposure in relation to T2DM incidence [35]. This finding is consistent with the results of many prospective studies, which reported a negative correlation between n-3 PUFA and the risk of T2DM in Chinese, but was not found in the US and Europe populations [41, 42]. The Asian subgroup in this study included not only East Asians but also Middle Eastern countries such as Iran, which may explain the different conclusions arised; more data on different ethnic groups are needed.

Our study did not show any change in insulin sensitivity and insulin resistance with fish oil supplementation among T2DM patients. Delicately, there was a meta-analysis [23] reported that fish oil had a positive effect on insulin sensitivity in people with metabolic disorders rather than healthy individuals and patients with clinically overt T2DM groups. Thus, fish oil supplementation may have a beneficial effect on patients with metabolic disorders or early diabetes rather than those with overt T2DM.

The major strength of this meta-analysis lies in the inclusion of only RCTs, which represent the gold study design to establish causality. Although some previous studies reported the association of fish oil and T2DM in observational studies, they cannot establish causality and they may be subject to confounding. The rigorous methodology we followed in this study also ensured high quality data. Some limitations should be acknowledged. Firstly, in the included RCTs, the ratio of EPA/DHA concentration in fish oil tended to be similar (i.e., around 3/2), so the study cannot assess whether different ratio of EPA/DHA can bring out discrepant results. Secondly, we considered to explore the variation trend of inflammatory factors related to T2DM with fish oil supplementation, which has been reported that fish oil has beneficial effects on T2DM through anti-inflammatory actions [10]. However, those data were available in few studies included in the meta-analysis.

## Conclusion

In conclusion, among patients with T2DM, fish oil supplementation leads to a favorable blood lipids profile (i.e., reduced TG levels and elevated HDL cholesterol levels), but does not improve glucose levels. Further investigation is needed to determine long term effects of fish oil supplementation and to understand the mechanisms (e.g., genomic variations) of heterogenous findings across populations.

## Data Availability

All data generated or analyzed during this study are included in this article.

## Abbreviations

CI: Confidence intervals
DHA: Docosahexaenoic acid
EPA: Eicosapentaenoic acid
FINS: Fast insulin
FPG: Fast plasma glucose
HbA1c: Glycosylated hemoglobin
HDL cholesterol: High density lipoprotein cholesterol
HOMA-IR: HOMA of insulin resistance
LDL cholesterol: Low density lipoprotein cholesterol
RCTs: Randomized controlled trials
SMD: Standard mean difference
T2DM: Type 2 diabetes mellitus
TC: Total cholesterol
TG: Triglyceride
